# Unicentric Castleman’s Disease with an Unusual Clinical Behavior

**DOI:** 10.7759/cureus.10973

**Published:** 2020-10-15

**Authors:** Sawsan A Aljubran, Basim F Khan, Maram M Alqahtani, Ahad Y Shaikh, Rizam A Alghamdi

**Affiliations:** 1 Family and Community Medicine, Imam Abdulrahman Bin Faisal University, Dammam, SAU

**Keywords:** castleman's disease, stomatitis, oral ulcers, genital ulcers

## Abstract

Castleman’s disease is a rare clinicopathological condition characterized by hyperplasia of lymph nodes. We report the case of a 12-year-old boy who presented with painful oral and genital ulcers, who was assumed to have Behçet’s disease. However, the patient did not show any clinical improvement on colchicine and systemic corticosteroids. Later, the patient developed severe abdominal pain and vomiting. Abdominal CT revealed a mass lesion superior to the right kidney. After a thorough investigation, he was diagnosed with unicentric Castleman’s disease. Despite the complete resection of the mass, the patient continued to have the symptoms of abdominal pain and orogenital ulcers. Immunomodulatory therapy resulted in remarkable clinical improvement. This case report demonstrates how unicentric Castleman’s disease can share similar clinical behavior to the multicentric disease.

## Introduction

Castleman’s disease is a group of idiopathic lymphoproliferative disorders that have different histological and prognostic characteristics from malignancy lymph node hyperplasia [1]. It was first described in 1956 by Castleman in a group of patients with benign lymphadenopathy [2]. Unicentric and multicentric Castleman’s disease are the two well-defined clinical subtypes. This disease has three histological subtypes: plasma cell type, the hyaline vascular type, and the mixed type variants [1]. The exact pathogenesis of Castleman’s disease remains unclear. However, several associations with viral infections, autoimmune disease, and malignancies have been reported [1,2].

Unicentric Castleman’s disease shows a slight female predominance, and the mean age for the diagnosis is 34 years. The optimal treatment for unicentric Castleman’s disease is complete surgical resection, which has an excellent prognosis, with an estimated 10-year survival rate of 95% [2].

## Case presentation

We report the case of a 12-year-old boy who was referred to the pediatric clinic at our institution with a history of painful oral and genital ulcers for one month before the presentation. The patient was seen by several general practitioners for stomatitis and was prescribed symptomatic treatment but showed no improvement. He had an unremarkable past medical history. No family history of autoimmune diseases was noted.

**Figure 1:**
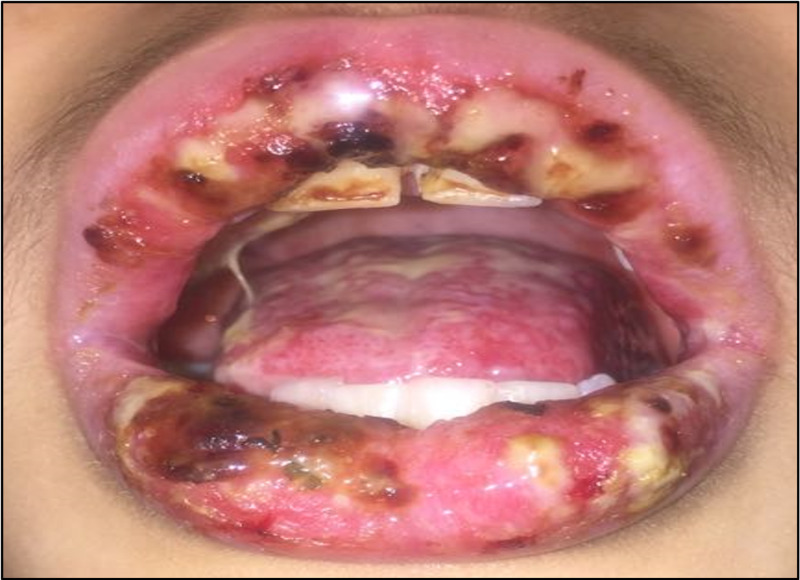
Multiple crusted hemorrhagic ulcers covering the buccal mucosa, palate, and gingiva with blackish lip crusts

Since the patient was not tolerating oral feeding, he was admitted for management and further investigation. He was started on broad-spectrum antiviral and antifungal therapy for two weeks. Considering that the patient had difficulty swallowing, he underwent an upper gastrointestinal endoscopy, which revealed severe gastroesophageal reflux disease with gastric and esophageal ulcers. Subsequently, he underwent colonoscopy, which had signs of mild colitis. In light of the clinical and endoscopic findings, a diagnosis of Behçet’s disease was made. Hence, the patient was started on colchicine and systemic corticosteroid for two weeks. No improvement was noted, and the patient lost around 15 kg since the disease onset. Further laboratory investigation revealed normal basic hematological and biochemical profiles. However, he had an elevated erythrocyte sedimentation rate (77 mm/hour) and mild elevation of fecal calprotectin. The viral serology panel, including Epstein-Barr virus, cytomegalovirus, and human herpesvirus 8, was normal. The patient’s condition progressed, and he developed multiple crusted hemorrhagic ulcers covering the oral mucosa (Figure 1).

**Figure 2:**
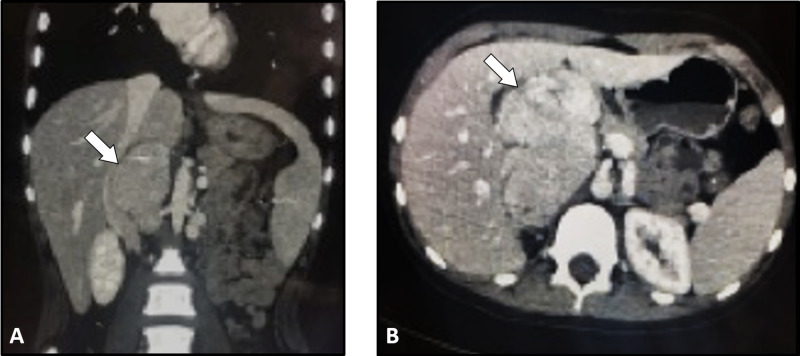
Abdominal CT images Coronal (A) and axial (B) CT images showing a mess lesion located superior to the right kidney. No evidence of thrombosis or lymph node enlargement is noted.

During the hospital course, the patient developed severe abdominal pain associated with nausea and vomiting. Abdominal ultrasonography revealed a large lobulated hyperechoic mass lesion compressing the gallbladder. An abdominal CT scan was performed for further evaluation of this mass. It demonstrated a mass lesion located superior to the kidney but was not crossing the midline. There was no evidence of thrombosis or lymphadenopathy (Figure 2).

**Figure 3:**
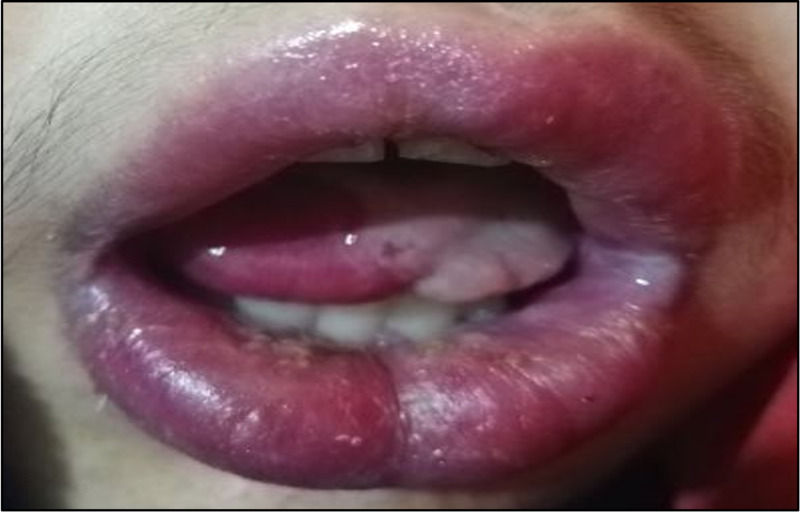
Remarkable improvement in the patient's condition after the systemic immunomodulatory therapy

Biopsies were taken from the buccal mucosa and the abdominal mass by an image-guided procedure. Histopathological examination of the buccal mucosa revealed a benign salivary gland with a sparse chronic inflammatory cell infiltrate. The specimen obtained from the abdominal mass showed a benign lymphoid tissue with no evidence of malignancy. The patient underwent laparotomy for the resection of the abdominal mass. Histopathological examination of the mass was consistent with the diagnosis of unicentric Castleman’s disease. Despite the surgical resection of the mass, the patient’s symptoms persisted. Subsequently, the patient was given intravenous methylprednisolone (20 mg/kg) for five days in addition to azathioprine, which showed remarkable improvement (Figure 3).

The medications were tapered gradually over a period of two weeks. The patient was discharged in a good condition on low-dose prednisolone and cyclosporine. He sustained remission for 18 months without any disease flare.

## Discussion

Castleman’s disease is a rare non-neoplastic lymphoproliferative disorder that is characterized by angiofollicular lymph node hyperplasia. It has two well-defined forms, which are the unicentric and multicentric types. The unicentric Castleman’s disease is usually localized and often does not need systemic therapy. On the other hand, multicentric Castleman’s disease is associated with a heterogenous group of symptoms and requires systemic therapy [1,2]. We reported a case of unicentric Castleman’s disease that shows a clinical behavior similar to the multicentric subtype.

The optimal treatment of the unicentric Castleman’s disease is a complete resection, with an excellent prognosis and survival. Radiotherapy is an alternative option if the lesion in unresectable [3]. Zhang et al. [4] conducted a multicentric study involving 121 patients with unicentric Castleman’s disease who underwent resection for the primary lesion and their outcome was excellent. In our case, however, the local resection did not achieve a resolution of the symptoms.

Pemphigus is a commonly associated disease with Castleman’s disease. It is considered as a paraneoplastic syndrome presenting with painful oral ulcers and polymorphic skin eruptions [5]. We have performed a buccal mucosa biopsy in the present case, but the histopathological examination was not consistent with paraneoplastic pemphigus.

Regarding the clinical manifestations of the disease, multicentric Castleman’s disease is found to have distinct clinical and demographic characteristics. Unlike unicentric Castleman’s disease, patients with the multicentric type are more likely to be older, having systemic symptoms (e.g., fever, weight loss, anorexia) and peripheral neuropathy and displaying abnormal laboratory findings such as anemia, hypoalbuminemia, leukocytosis, and elevated creatinine [6,7].

Unlike the unicentric subtype, the optimal treatment method for the multicentric subtype is not well established. The treatment approaches for the multicentric subtype were adapted from the management strategies for lymphoma and multiple myeloma. Such agents include systemic corticosteroids, intravenous immunoglobulins, immunomodulators (e.g., azathioprine), and recently the biologic agents. The multicentric subtype has a worse prognosis than the unicentric subtype, with an overall five-year survival rate of 51.2% [4]. Certain clinical and demographic factors were found to have been associated with unfavorable outcomes in patients with the multicentric Castleman’s disease. These factors include advanced age and splenomegaly [4].

## Conclusions

Castleman’s disease is a very uncommon lymphoproliferative disorder that could pose a diagnostic challenge. While surgical resection of the mass lesion in the unicentric Castleman’s disease is often curative, it should be kept in mind that some cases of the unicentric subtype share a similar clinical behavior as the multicentric subtype. Multidisciplinary input is crucial in the management of this disease.
